# MiR‐129‐5p promotes docetaxel resistance in prostate cancer by down‐regulating *CAMK2N1* expression

**DOI:** 10.1111/jcmm.14050

**Published:** 2019-12-26

**Authors:** Cheng Wu, Chunqing Miao, Qingsheng Tang, Xunrong Zhou, Pengshan Xi, Ping'an Chang, Lixin Hua, Haodong Ni

**Affiliations:** ^1^ Department of Urology The First Affiliated Hospital of Nanjing Medical University Nanjing Jiangsu China; ^2^ Department of Urology People's Hospital of Dongtai City Dongtai Jiangsu China

**Keywords:** *CAMK2N1*, docetaxel resistance, MiR‐129‐5p, prostate cancer

## Abstract

This study focuses on the effect of miR‐129‐5p on docetaxel‐resistant (DR) prostate cancer (PCa) cells invasion, migration and apoptosis. In our study, the expression of *CAMK2N1* was assessed by qRT‐PCR in PCa patient tissues and cell lines including PC‐3 and PC‐3‐DR. Cells transfected with miR‐129‐5p mimics, inhibitor, *CAMK2N1* or negative controls (NC) were used to interrogate their effects on DR cell invasions, migrations and apoptosis during docetaxel (DTX) treatments. The apoptosis rate of the PCa cells was validated by flow cytometry. Relationships between miR‐129‐5p and *CAMK2N1* levels were identified by qRT‐PCR and dual‐luciferase reporter assay. *CAMK2N1* was found to be down‐expressed in DR PCa tissue sample, and low levels of *CAMK2N1* were correlated with high docetaxel resistance and clinical prediction of poor survival. *CAMK2N1* levels were decreased in DR PCa cells treated with DXT. We further explored that up‐regulation of miR‐129‐5p could promote DR PCa cells viability, invasion and migration but demote apoptosis. Involved molecular mechanism studies revealed that miR‐129‐5p reduced downstream *CAMK2N1* expression to further impact on chemoresistance to docetaxel of PCa cells, indicating its vital role in PCa docetaxel resistance. Our findings revealed that miR‐129‐5p contributed to the resistance of PC‐3‐DR cells to docetaxel through suppressing *CAMK2N1* expression, and thus targeting miR‐129‐5p may provide a novel therapeutic approach in sensitizing PCa to future docetaxel treatment.

## 1 INTRODUCTION

Prostate cancer (PCa), one of the most common cancers among males, is the second‐leading cause of cancer‐related male deaths in America, and its occurrence is strikingly increasing in China.^1^ After initial successful treatment by androgen deprivation therapy, most of the patients will eventually progress to castration‐resistant prostate cancer (CRPC), which, as the lethal form of PCa, is often incurable nowadays.^2^ So far, the most commonly prescribed first‐line therapy for CRPC is docetaxel, which is believed to offer symptomatic and survival benefits in men with metastatic hormone‐refractory PCa. However, over time cancer cells develop resistance to docetaxel and prostate tumour growth will again proceed regardless of the presence of the drug. Resistance can be formed through a variety of mechanisms, both intrinsic and extrinsic, but the details remain largely unknown.^3^

MicroRNAs (miRNAs) are small 19‐25 nucleotide‐long, single‐stranded non‐coding RNAs that silence target genes by cleaving mRNA molecules or inhibiting translation.^4^ Recently, studies have shown that miRNAs are frequently misregulated in many types of human cancers. For instance, some may act as potent oncogene promoting tumour growth and migration but demoting apoptosis, while others may act as tumour suppressor genes by targeting downstream genes to inhibit migration and invasion of tumour cells.^5^ Among various miRNAs, miR‐129 has been shown to play a key role in tumourigenesis, tumour progression, chemotherapy resistance and cell proliferation.^6^ For instance, miR‐129 was initially confirmed to be decreased in undifferentiated gastric cancer tissue, colorectal, gastric and liver cancer.^7^ In oesophageal neoplasms, there have been conflicting studies concerning miR‐129 expression, with some groups indicating it was down‐regulated compared to normal tissue,^8^, ^9^ while others claiming it was increased in tumour tissue.^10^ Besides, miR‐129‐3p was reported to be a novel metastatic microRNA in PCa cells.^11^ Collectively, these reports suggested that the function of miR‐129 is highly tumour specific. Besides, researchers found miR‐129‐3p inhibited docetaxel‐induced apoptosis of breast cancer cells by down‐regulation of the CP110 protein.^12^ However, the involvement of miR‐129 in the chemoresistance of cancer, especially in PCa, is largely unknown. Hence, a thoroughgoing understanding of these miscellaneous functions would be indispensable for further steps at developing promising therapies.

*CaMKII*, which belongs to the calcium/calmodulin‐dependent protein kinase II family, is a serine/threonine‐specific protein kinase and can phosphorylate nearly 40 distinctive proteins, among which are kinases, ion channels and transcription factors.^13^, ^14^ By activating *MEK/ERK*,* CAMKII* cascade enhances the phosphorylation of p27^Kip1^ to control cell‐cycle.^13^ There are two potent and specific inhibitors of *CAMKII*, which have been characterized in human, including *CAMK2N1* (also known as *CANK2N*α) and *CAMK2N*β genes. The *CAMK2N*β was the first discovered human *CAMKII* inhibitor and was cloned from human dendritic cells, with an inhibitory effect on the growth of colon adenocarcinoma LoVo cells.^14^ The *CAMK2N1* is composed of 78‐amino acids and was initially identified in the cell junction and synapse.^13^, ^14^, ^15^ Additionally, *CAMK2N1*‐mediated inhibition of CaMKII activity controls the progress of cell cycle in colon cancers through deactivation of MEK/ERK kinase activity and p27 protein accumulation.^13^, ^14^ In a recent study, genome‐wide gene expression analysis uncovered that *CAMK2N1* regulated the expression of pivotal genes related to cell‐cycle control and apoptosis.^15^ Researchers also showed that *CAMK2N1* has a vital role to affect tumourigenesis and tumour development.^13^, ^14^ Previous study revealed that its expression was down‐regulated in PCa, and reintroduction of *CAMK2N1* remarkably impaired human PCa cell proliferation and in vivo tumour growth.^15^ Furthermore, researchers found that *CAMK2N1* played a suppressive role in castration‐resistance PCa via suppressing androgen receptor mRNA expression and its regulator.^15^ Together, these data indicate that *CAMK2N1* plays a pivotal role in the progression of PCa. However, for *CAMK2N1*, its complete and detailed molecular mechanisms, such as upstream pathway and functions and whether it related to other types of drug resistance in PCa are still unclear.

Here, we analysed microarray data and screened out *CAMK2N1* as one of the most down‐regulated mRNAs in docetaxel‐resistant (DR) PCa cells. The biological function of *CAMK2N1* was comprehensively investigated in vitro, exhibiting that *CAMK2N1* can effectively inhibit docetaxel resistance in PCa cells. We further employed in silico analysis and molecular techniques to confirm that *CAMK2N1* is the target of miR‐129‐5p, which significantly rescued miR‐129‐5p promoted PCa docetaxel resistance.

## 2 MATERIALS AND METHODS

### 2.1 Patient samples

Thirty‐six PCa tissues were obtained from docetaxel‐free PCa patients (n = 18) and DR PCa patients (n = 18) by radical prostatectomy at the First Affiliated Hospital of Nanjing Medical University in Jiangsu, China. All the patients were confirmed by exhaustive diagnosis and the tissue samples were independently interrogated by three experienced pathologists. All samples were collected with the informed consent of the patients and the study was approved by the local ethical committee.

### 2.2 Microarray

Affymetrix microarray platform GPL570 and microarray data GSE33455 used for validation were obtained from Gene Expression Omnibus database (http://www.ncbi.nlm.nih.gov/geo/). This dataset included 12 prostate tissues samples, three DU‐145 cell lines, three DR DU‐145 cell lines, three PC‐3 cell lines and three PC‐3‐DR cell lines. The threshold used to screen up‐regulated and down‐regulated mRNA was log_2_ (FC) >1 and log_2_ (FC) <−1 (*P* < 0.05) respectively.

### 2.3 Cell culture, reagents and materials

Human embryonic kidney cell line HEK293T and human PCa cells PC‐3 were obtained from BeNa Culture Collection (Beijing, China), and maintained in Dulbecco's Modified Eagle's Medium (DMEM; Sigma‐Aldrich, St. Louis, MO, USA) with 10% (v/v) foetal bovine serum (Invitrogen, CA, USA) and 1% (v/v) penicillin‐streptomycin (Sigma‐Aldrich). The docetaxel‐resistant PC (PC‐3‐DR) cells were created by culturing parental PC‐3 cells in gradually increasing concentrations of docetaxel (Sangon Biotech, Shanghai, China) and thereafter maintained in 60 nmol/L docetaxel‐containing media for 10 months. All cells were replaced medium and passaged 2‐3 times per week and cultured at 37°C in a humidified atmosphere consisting of 5% CO_2_ in incubator.

### 2.4 RNA extraction and qRT‐PCR

Trizol Reagent (Invitrogen) was used to extract total RNA including miRNA. Next, 1 μg of total RNA per sample was converted to cDNA by accurate primers using the PrimeScript^™^ RT‐PCR Kit (TAKARA, Japan) for the detection of CAM2NK1. cDNAs were amplified using PrimeSTAR^®^ HS DNA Polymerase (TAKARA). Quantitative reverse transcription PCR (qRT‐PCR) was conducted using the THUNDERBIRD SYBR^®^ qPCR Mix (Toyobo, Japan). The relative quantification value of mRNAs was normalized to control and calculated through the 2^−ΔΔCt^ method. The GADPH and U6 were used as internal control genes for mRNA and miRNA respectively. Primer sequences are given in (Table 1).

**Table 1:** Primer sequences

Gene	Sequences: 5′→3′
CAMK2N1	
Forward	GCAAGCGGGTTGTTATTGAAGA
Reverse	GGTTGTTGATTTCATCGTGGGT
GAPDH	
Forward	CTATAAATTGAGCCCGCAGCC
Reverse	GCCCAATACGACCAAATCCGT
MiR‐129‐5p	
Forward	GGGGGCTTTTTGCGGTCTGG
Reverse	AGTGCGTGTCGTGGAGTC
U6	
Forward	CTCGCTTCGGCAGCACA
Reverse	AACGCTTCACGAATTTGCGT
CAMK2N1 cDNA	
Forward	GAATTCATGTCGGAGGTGCTGC
Reverse	CTCGAGTTAGACACCAGGAGGT

### 2.5 Western blot analysis

Protein was extracted from tissues using RIPA buffer. After denaturing at 95°C for 10 minutes, protein was separated on a 12% SDS‐polyacrylamide gel and then transferred to a polyvinylidene fluoride membrane. After blocking with TBST buffer containing 5% non‐fat milk for 1 hour at room temperature, the membrane was co‐incubated with primary antibodies: rabbit anti‐CAMK2N1 (PA5‐23740, 1:1000; Thermo Fisher Scientific, Waltham, MA, USA), rabbit anti‐p‐ERK1/2 (ab223500, 1:400; Cambridge, MA, USA), rabbit anti‐ERK1/2 (ab17942, 1:1000; Abcam), rabbit anti‐p‐EMK (ab60002, 1:500; Abcam), rabbit anti‐EMK (ab60002, 1:2000; Abcam), rabbit anti‐Bax (ab32503, 1:1000; Abcam), rabbit anti‐Bcl2 (Bcl2, 1:2000; Abcam) and GAPDH (ab8245, 1:500; Abcam) at 4°C overnight. The membrane was co‐incubated with HRP‐conjugated goat anti‐rabbit IgG (ab6721, 1:2000; Abcam) at 37°C for 1 hour after washing with TBST. Finally, the results were visualized using Chemical Mp Imaging System (Bio‐Rad, Hercules, CA, USA) and analysed by Gel‐ProAnalyzer (UnitedBio, USA). Protein levels were normalized to GAPDH.

### 2.6 Cell transfection

Twenty‐four hours before transfection, 1 × 10^6^ cells/well logarithmic growth phase PD‐3 or PC‐3‐DR cells were cultured until reaching 80%‐90% confluency in six‐well plates. The miR‐129‐5p mimics, inhibitors and negative control (NC) were synthesized by Sangon Biotech. MiRNA‐129‐5p mimics, inhibitors and NC were transfected into PC‐3 and PC‐3‐DR cells employing Lipofectamine™ 3000 (Invitrogen) according to the manufacturer's protocol. The final concentration of all constructs in the transfection system was optimized to 20 μmol/L. *CAMK2N1* was amplified by PCR using PC‐3 cell lines as templates. The constructs were transfected into PC‐3 or PC‐3‐DR cells using Lipofectamine™ 3000. After 24, 48 or 72 hours, the cells were harvested for following analyses.

### 2.7 Cell proliferation assay

Two thousand PC‐3 and PC‐3‐DR cells were cultured in 96‐well plates each hole and 10 μL of CCK‐8 solution (Beyotime, Shanghai, China) was added to each well at 24, 48 or 72 hours after transfection. Cells were maintained for another 4 hours at 37°C in a 5% CO_2_ incubator. The optical density was read at 450 nm with a SpectraMax i3x Multi‐Mode Detection Platform (Molecular Devices, USA). The resistance value of docetaxel was calculated as the ratio of the 50% inhibitory concentration (IC_50_) for PC‐3‐DR divided by the IC50 for wild‐type PC‐3; the IC_50_ values were verified in vitro following the Cell‐Counting kit 8 (CCK8) (Beyotime).

### 2.8 Flow cytometry

Cell apoptosis was detected by Annexin V‐PE Apoptosis Detection Kit (Beyotime). Cells were added in a six‐well plate until grown to 60%‐80% confluence before transfection. Twenty‐four hours after the transfection, 60 nmol/L cisplatin was added to the medium. After 48 hours, cells were washed twice in PBS (Sangon Biotech) before resuspension in 500 μL 1× Binding Buffer. Next, cells were mixed with 5 μL of Annexin V‐FITC and 5 μL of propidium iodide in darkness at 37°C for 20 minutes, followed by adding 1× Binding Buffer to each tube. Stained cells were measured by flow cytometer (Bio‐Rad) using Cell Quest Pro software (BD, USA). The data were analysed via FlowJo9.1 software.

### 2.9 Transwell assay

Matrigel gel (BD, USA) was used to coat 24‐well Transwell plates (Millipore, Beijing, China). At 48 hours after transfection, cells (4 × 10^4^ cells each well) were plated into the upper chambers of Transwell plates and the medium supplemented with 10% FBS was plated into the lower chambers. The membrane was fixed in methanol, and then stained using haematoxylin after 24 hours of incubation. Stained cells were visualized and counted under a microscope (200 magnification), The cell invasion was detected by Transwell assay using Matrigel Invasion Chambers (BD, USA).

### 2.10 Dual‐luciferase reporter assay

The 3′‐UTR of human *CAMK2N1* was amplified by PCR and then cloned into the pMIR‐REPORT™ miRNA expression reporter vector (Ambion, TX, USA), obtaining the *CAMK2N1* 3′‐UTR wildtype (WT) firefly luciferase reporter gene. We performed overlap PCR and introduced mutations into the seed sequences of all four predicted miR‐129‐5p target sites within the *CAMK2N1* 3′‐UTR and generated the *CAMK2N1* 3′‐UTR mutant (MT). Similarly, the *CAMK2N1* 3′‐UTR MT was digested and ligated to the multi‐cloning sites of the pMIR‐REPORT miRNA expression reporter plasmid. All the recombinant DNAs were verified by DNA sequencing. HEK293T cells were inoculated onto 24‐well plates and co‐transfected with luciferase reporter constructs containing the wild‐type or mutant *CAMK2N1* 3′‐UTR firefly luciferase reporters, pRL‐TK and miR‐129‐5p mimics or mimics control using Lipofectamine 3000. Luciferase activities were detected 48 hours after the transfection by the dual‐luciferase Reporter Assay System (Promega, WI, USA). Firefly luciferase activity was normalized to renilla luciferase activity.

### 2.11 Statistical analysis

All quantitative values were presented as the mean ± SD of at least three repeated individual experiments for each group and they were statistically analysed with one‐way ANOVA. All of the statistical analyses were made using GraphPad Prism v6.0 (GraphPad Software, Inc.) and SPSS (version 21.0). A value of *P* < 0.05 was considered statistically significant.

## 3 RESULTS

### 3.1 CAMK2N1 is down‐regulated in PCa DR tumour tissues and cell lines

**Figure 1:**
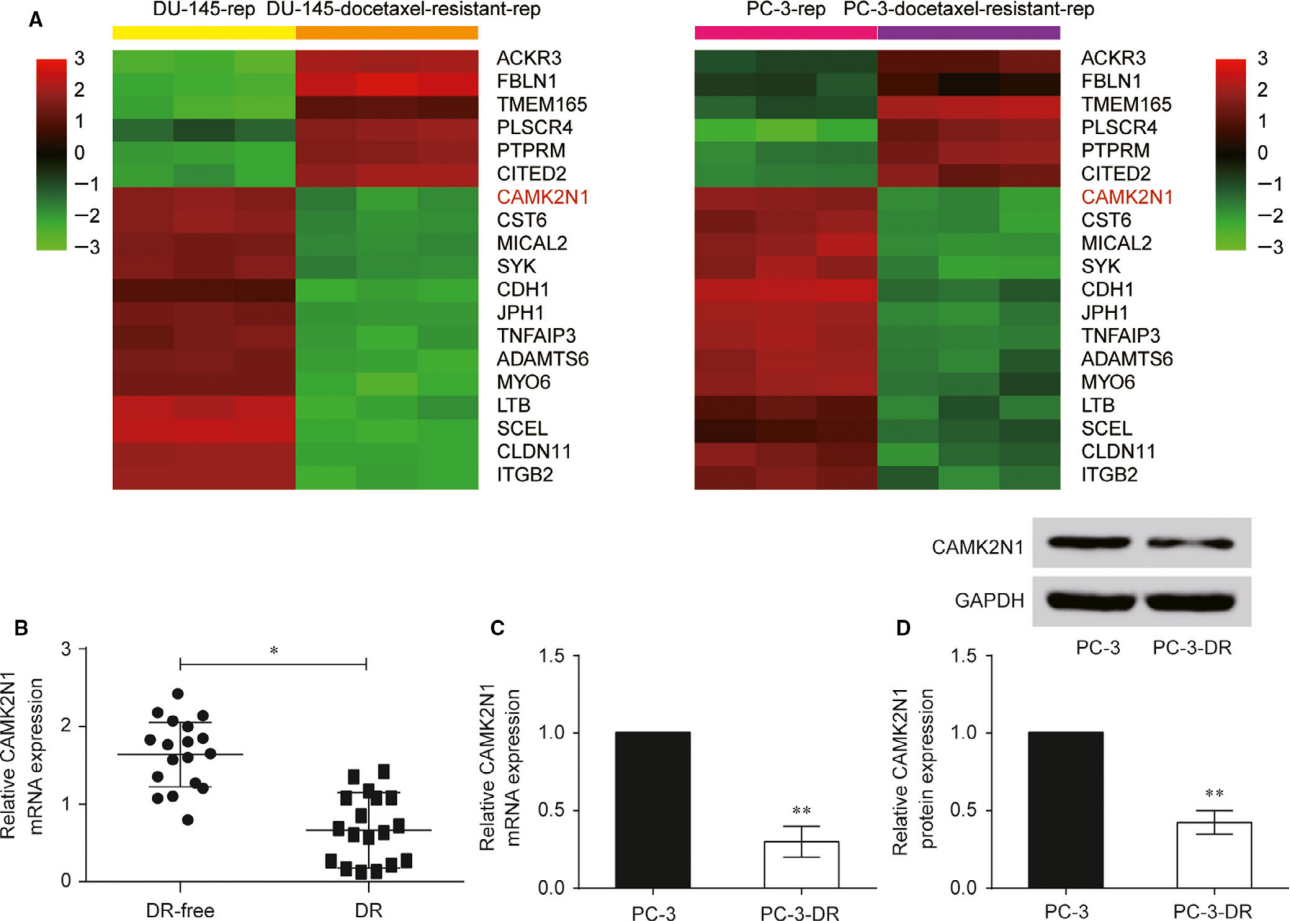
Differential expression of *CAMK2N1* in prostate cancer resistant cell lines. (A) Heat map of differentially expressed mRNAs in normal and docetaxel‐resistant (DR) cell lines (DU‐145 and PC‐3). The colour gradation indicates the log of fold change (FC) with a base of 2. (B) The relative mRNA expression levels of *CAMK2N1* in 18 pairs of docetaxel‐free and DR tissues of prostate cancer patients were detected by qRT‐PCR. Data are expressed as mean ± SD (n = 36). *P < 0.05 compared to the docetaxel‐free group. (C, D) The relative mRNA and protein expression levels of *CAMK2N1* in prostate cancer cell line PC‐3 and PC‐3 DR cell line (PC‐3‐DR) were detected by qRT‐PCR and western blot. Data are expressed as mean ± SD (n = 36). **P < 0.01 compared to the PC‐3 group

Based on microarray platform GPL570 and microarray data GSE33455, we used a *t* test (*P* < 0.05) combined with fold change (FC) log_2_ (FC) >1 and log_2_ (FC) <−1 for up‐regulated and down‐regulated mRNAs respectively. The fold change log_2_ (FC) >|1| had been defined as a screening threshold to ascertain differentially expressed mRNA. The six up‐regulated and 13 down‐regulated mRNAs in DR PC‐3 cells compared with that in normal PC‐3 cells were reflected by heat map (Figure 1A). To explore the relationship between *CAMK2N1* and docetaxel resistance of PCa cells, we assessed the expressions of *CAMK2N1* mRNA in 18 tissues of docetaxel‐free PCa patients and 18 tissues of DR PCa patients. As shown in Figure 1B, an inverse correlation of *CAMK2N1* expression and docetaxel resistance of PCa was observed in all samples. Next, we confirmed that mRNA and protein level of CAMK2N1 were down‐regulated in DR PCa cell lines (Figure 1C,D). In short, these data indicate *CAMK2N1* may have molecular and cellular functions in docetaxel resistance of PCa cells. Thus, *CAMK2N1* was chosen for further research.

### 3.2 The docetaxel‐resistance of PC‐3‐DR is significantly stronger than PC‐3

**Figure 2:**
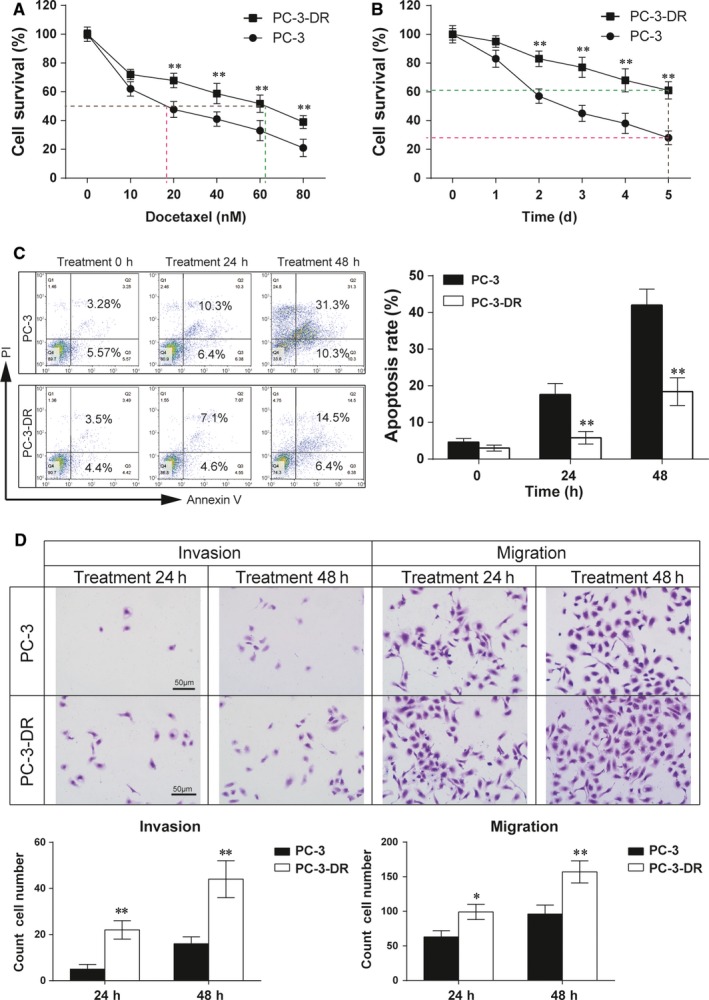
Increased cell viability and decreased apoptosis of PC‐3‐DR cells in docetaxel treatment. (A) Cells were cultured in 10% FBS media treated with different concentrations (from 0 to 80 nmol/L) of docetaxel. IC_50_ of docetaxel was determined after 6 days of treatment. Data are expressed as mean ± SD (n = 5). **P < 0.01 compared to the PC‐3 group. (B) Dynamic changes of cell activity during and after docetaxel treatment. 60 nmol/L docetaxel was used. Cell survival rate was measured at days 0, 1, 2, 3, 4 and 5, respectively. Data are expressed as mean ± SD (n = 5). **P < 0.01 compared to the PC‐3 group. (C) Cells were treated with docetaxel (60 nmol/L). Flow cytometry analyses show apoptosis rate in both PC‐3 and PC‐3‐DR cell lines after docetaxel (60 nmol/L) treatment. Data are expressed as mean ± SD (n = 5). **P < 0.01 compared to the PC‐3 group. (D) Transwell assay was performed to detect cell migration and invasion ability after DTX treated for 24 h, scale bar = 50 μm. Results are expressed as mean ± SD (n = 5). *P < 0.05, **P < 0.01 compared to the PC‐3 group

We next studied the cell viability and apoptosis between PC‐3 cells and experimentally generated DR sublines (PC‐3‐DR). CCK‐8 was used to detect the cell viability of PC‐3 and PC‐3‐DR under different concentrations of docetaxel. As shown in Figure 2A (*P* < 0.01), the cell viability of PC‐3‐DR is significantly higher than PC‐3 cells during docetaxel treatment, with the IC50 of PC‐3‐DR cells is 61.2 nmol/L compared to 18.9 nmol/L in PC‐3 cells. Therefore, PC‐3‐DR cells revealed higher docetaxel resistance than PC‐3 cells. In order to testify the time‐dependent effects of docetaxel on cell viability, we add 60 nmol/L docetaxel to PC‐3 and PC‐3‐DR cells. The results show that at the fifth day during docetaxel treatment, PC‐3‐DR cells have a survival rate over 60% while PC‐3 cells nearly die out (Figure 2B, *P* < 0.01), using flow cytometry to investigate apoptosis of PC cells, we found that after 24 hours of docetaxel treatment, the apoptosis rate of PC‐3‐DR cells is significantly lower than PC‐3 cells (Figure 2C, *P* < 0.01). Transwell assay was used to explore the ability of cell invasion and migration, it shows that the invasion and migration ability of PC‐3‐DR are significantly higher than PC‐3 cells (Figure 2D, *P* < 0.01). Altogether, PC‐3‐DR exhibited faster growth (Figure 2A,B), stronger apoptotic resistance (Figure 2C), invasion and migration ability (Figure 2D) in the docetaxel‐treated medium compared with PC‐3. In brief, PC‐3‐DR cells are more DR than PC‐3 cells. Therefore, PC‐3‐DR cells were used for the following trials.

### 3.3 Expression of CAMK2N1 attenuated docetaxel resistance PC‐3‐DR cells

**Figure 3:**
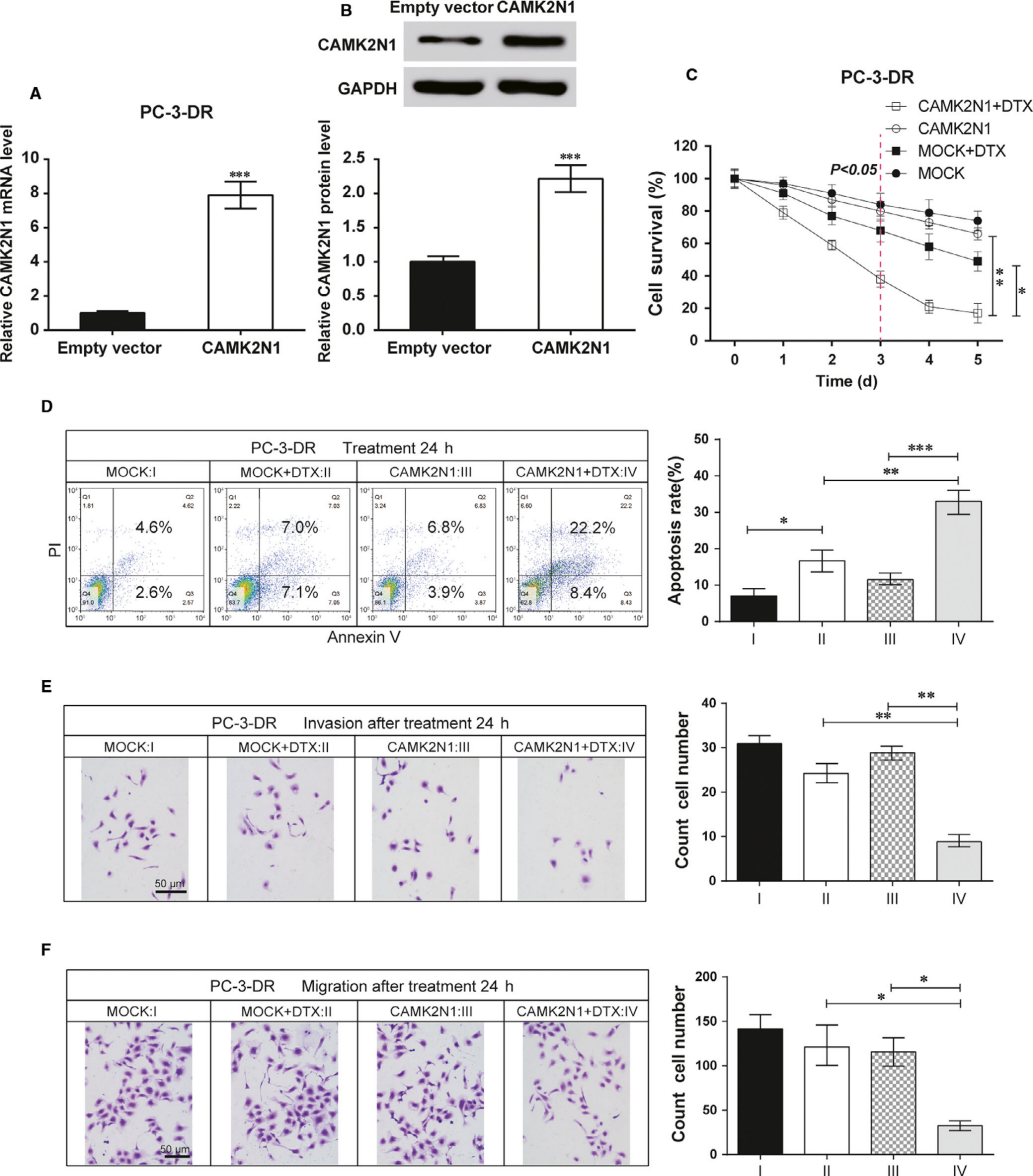
*CAMK2N1* inhibited docetaxel resistance in prostate cancer (PCa). (A, B) After *CAMK2N1* overexpression vector (pcDNA 3.1‐*CAMK2N1*) transfection, *CAMK2N1* mRNA expression was determined by qRT‐PCR and western blot in PC‐3‐DR cells. Data are expressed as mean ± SD (n = 4). **P < 0.01 compared to the control group. (C) Cell survival rate of PC‐3‐DR treated with DTX was detected by CCK‐8 assay at days 0, 1, 2, 3, 4 and 5, respectively. (D) Apoptotic cell death was measured with flow cytometry analyses after transfection with pcDNA 3.1‐*CAMK2N1* and treatment of DTX for 24 h. *P < 0.05, **P < 0.01, ***P < 0.001. (E, F) PCa cells invasion and migration ability were detected after treatment of DTX for 24 h, scale bar = 50 μm. The cell numbers were counted and results are expressed as mean ± SD (n = 5). **P < 0.01 compared to the PC‐3 group

We next explored the role of *CAMK2N1* in the docetaxel resistance of PC‐3‐DR cells. We constructed *CAMK2N1* expression vectors and transfected it into PC‐3‐DR cells. The expression level of *CAMK2N1* was validated using qRT‐PCR and western blot after CAMK2N1 vector was transfected to PC‐3‐DR cells (Figure 3A,B, *P* < 0.01). Cell proliferation ability was assayed by the CCK‐8 kit as described above. The proliferation rate of PC‐3‐DR cells ectopically expressed *CAMK2N1* under 60 nmol/L docetaxel treatment was markedly reduced when compared to that of mock group (Figure 3C, *P* < 0.01). And the apoptotic analyses revealed that up‐regulation of *CAMK2N1* effectively decreased docetaxel resistance in PC‐3‐DR cells (Figure 3D, *P* < 0.01). Additionally, the *CAMK2N1* overexpressing PC‐3‐DR cells exhibited impaired cell invasion and migration abilities (Figure 3E,F). Collectively, these data indicate that overexpression of *CAMK2N1* may enhance the docetaxel sensitivity in PC‐3‐DR cells.

### 3.4 CAMK2N1 is a target of miR‐129‐5p in PCa cells

**Figure 4:**
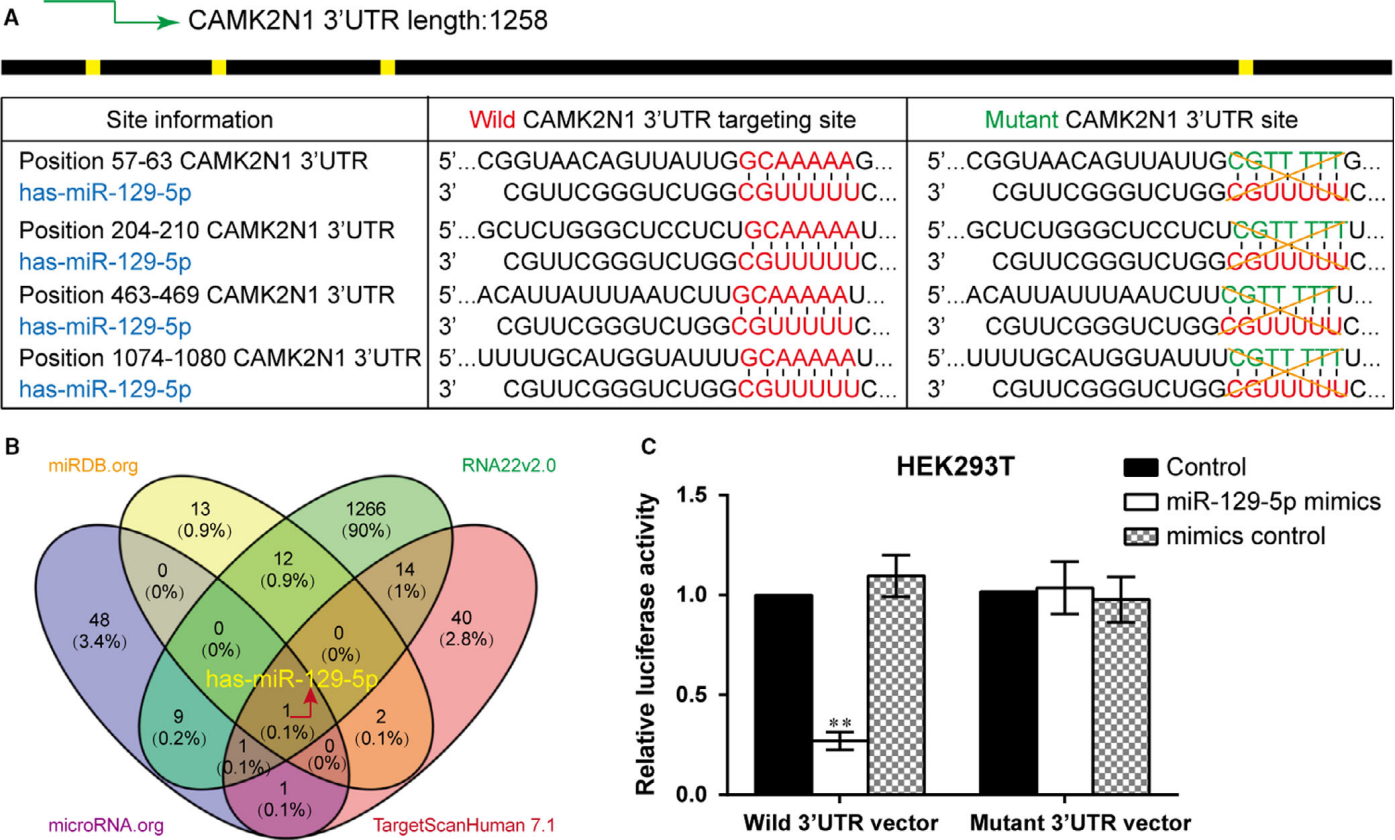
MiR‐129‐5p targeted *CAMK2N1* 3′UTR. (A) Base pairing complement suggested the putative miR‐129‐5p binding position at 3′‐UTR of *CAMK2N1*. (B) Venn diagram displayed miR‐129‐5p shared in the databases of miRDB, microRNA, RNA22v2.0 and TargetScan Human 7.1. (C) Dual‐luciferase reporter assays verified just miR‐129‐5p has the target‐relationship to *CAMK2N1* 3′UTR. Data are expressed as mean ± SD (n = 3). **P < 0.01 compared to the co‐transfected of wild *CAMK2N1* 3′UTR vector and transfection reagent group

Next, we were eager to find upstream regulator candidates that could mediate the *CAMK2N1*‐induced docetaxel‐sensitive to docetaxel in PC‐3‐DR cells. We included four miRNA databases including miRDB, miRBase, RNA22v2.0 and TargetScan Human 7.1 (Table S1) to explore the potential miRNA which may regulate *CAMK2N1*‐induced docetaxel resistance. We predicted that miR‐129‐5p may potentially target on four sites of 3′‐UTR of *CAMK2N1* (Figure 4B). To ascertain whether *CAMK2N1* is fine‐tuned by miR‐129‐5p in PC‐3‐DR cells, we used PCR mutagenesis approach to mutate four sites of 3′‐UTR of *CAMK2N1* and constructed (Figure 4A) the *CAMK2N1* 3′UTR WT and *CAMK2N1* 3′UTR MT (shown in Section 2), and placed them downstream of the luciferase reporter gene, *CAMK2N1* 3′‐UTR WT and *CAMK2N1* 3′‐UTR MT respectively. In HEK293T cells, ectopically expressed miR‐129‐5p could only impair the normalized luciferase activity with the wild‐type 3′UTR of *CAMK2N1*, but not with the mutant‐type (Figure 4C). These data revealed that miR‐129‐5p can directly target *CAMK2N1* expression in vitro.

### 3.5 MiR‐129‐5p strengthens cell viability and inhibits apoptosis of PCa cells via down‐regulating CAMK2N1

**Figure 5:**
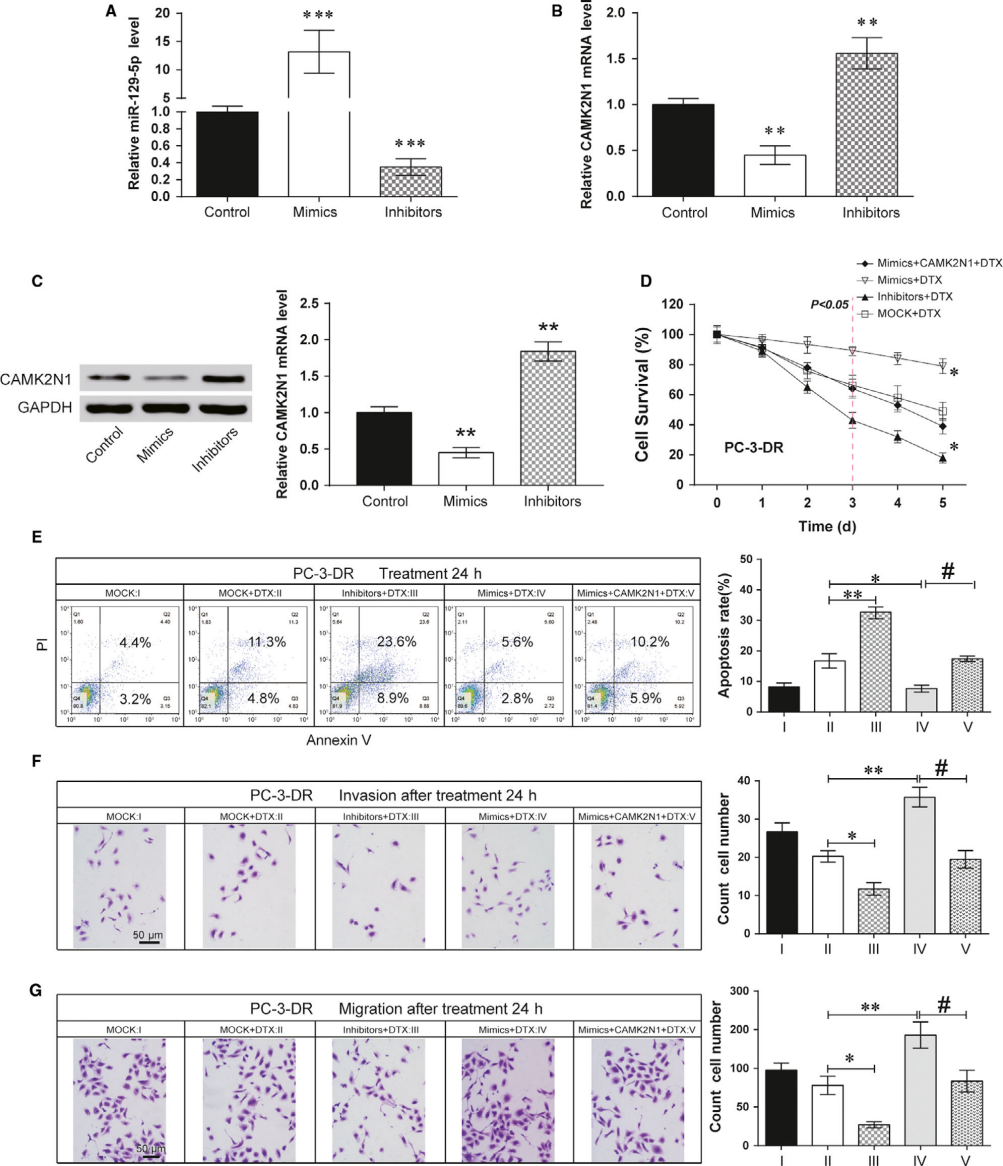
MiR‐129‐5p promoted the drug resistance of docetaxel in PCa by targeting *CAMK2N1*. (A) miR‐129‐5p mimics and inhibitors expressed in PC‐3‐DR cells. Data are expressed as mean ± SD (n = 4). **P < 0.01 compared to the control group. (B) Effects of miR‐129‐5p mimics and inhibitors on *CAMK2N1* mRNA was detected by qRT‐PCR. Data are expressed as mean ± SD (n = 4). **P < 0.01 compared to the control group. (C) Effects of miR‐129‐5p mimics and inhibitors on CAMK2N1 protein was detected by western blot. Data are expressed as mean ± SD (n = 4). **P < 0.01 compared to the control group. (D) After treated PC‐3‐DR cell with DTX (60 nmol/L) for 24 h, miR‐129‐5p promoted cell survival of PC‐3‐DR while *CAMK2N1* inhibited the positive effect of miR‐129‐5p on cell survival. *P < 0.05, compared with MOCK+DTX group. (E) MiR‐129‐5p attenuated apoptosis of PC‐3‐DR while *CAMK2N1* promoted cell apoptosis. *P < 0.05, **P < 0.01, compared with MOCK+DTX group; #*P* < 0.05, compared with mimics+DTX group. (F, G) MiR‐129‐5p facilitated the invasion and migration of PC‐3‐DR while *CAMK2N1* impeded the positive effect of miR‐129‐5p on cell invasion and migration, scale bar = 50 μm. *P < 0.05, **P < 0.01, ***P < 0.001 compared with MOCK+DTX group; #*P* < 0.05, compared with mimics+DTX group

**Figure 6:**
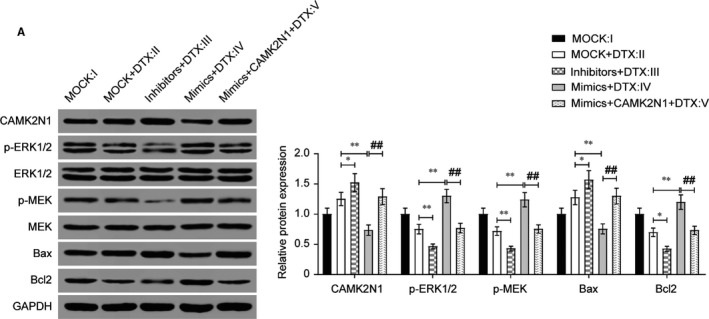
CAMK2N1 reversed the effect of miR‐129‐5p on inhibiting activation of ERK/MEK and promoting Bax/Bcl2. (A) Expression levels of CAMK2N1, p‐ ERK1/2, ERK1/2, p‐MEK1, MEK1, Bcl‐2 and BAX were determined by Western blot in PC‐3‐DR cells. *P < 0.05, **P < 0.01, compared with MOCK+DTX group; ##*P* < 0.01, compared with mimics+DTX group

To evaluate the involvement of miR‐129‐5p in regulation of docetaxel sensitivity via *CAMK2N1*, we transiently transfected miR‐129‐5p mimics, inhibitors and NC into PCa cell lines. As shown in Figure 5A (*P* < 0.01), qRT‐PCR results demonstrated miR‐129‐5p mimics ectopically expressed in PC‐3‐DR cells. Moreover, the *CAMK2N1* mRNA and protein level were effectively impaired by miR‐129‐5p mimics (Figure 5B,C, *P* < 0.01). We keep these transfected cells in docetaxel (60 nmol/L) or routine culturing conditions for 3 days. We compared the survival rate in these cells (Figure 5D, *P* < 0.05), miR‐129‐5p mimics expressed PC‐3‐DR cells consistently demonstrated an obviously higher survival rate which indicated improved docetaxel resistance, while overexpressed *CAMK2N1* totally suppressed this tendency. To verify whether the effects of miR‐129‐5p on cell proliferation were caused by attenuation of apoptosis, we performed an apoptosis assay by flow cytometry. We transfected PC‐3‐DR cells with 20 uM miR‐129‐5p mimic, inhibitors or NCs for 24 hours and then treated these cells with docetaxel (60 nmol/L) for another 24 hours. Our results suggested that miR‐129‐5p expression attenuated docetaxel‐induced cellular apoptosis while *CAMK2N1* completely reversed this trend (Figure 5E, *P* < 0.05). Similarly, via Transwell assay, we found that overexpression of *CAMK2N1* markedly inhibited miR‐129‐5p induced increase in PC‐3‐DR cell migration and invasion compared with mock transfection in docetaxel treatment (Figure 5F,G, *P* < 0.05). Furthermore, we performed western blot analyses to analyse the expression of a subset of related protein (Figure 6). Compared with mock transfection in docetaxel treatment overexpression of miR‐129‐5p in PC‐3‐DR cells resulted in decreased CAMK2N1 and Bax protein expression and increased p‐ERK1/2, p‐MEK and Bcl2 protein expression, which was reversed by overexpression of CAMK2N1. These results suggested the important role of *CAMK2N1* in cell invasion and migration of PC‐3‐DR cells regulated by miR‐129‐5p. Besides, PC‐3‐DR cells with miR‐129‐5p overexpression were clearly more resistant to docetaxel treatment.

## 4 DISCUSSION

As docetaxel is widely used in chemotherapy for various types of cancer, including PCa, improvements of chemosensitization strategies will have crucial clinical implications. Accumulating evidence supports the concept that miRNAs are pivotal handlers of drug resistance and consequently, that modulation of their activities could be a promising therapeutic strategy for they are characteristics of regulating gene expression and participating in gene regulatory networks by sequence‐specific binding to their target mRNAs. Here, we first employed in silico analysis and identified *CAMK2N1* as one of the most down‐regulated genes in DR PCa cells. Next, the biological function of *CAMK2N1* in docetaxel resistance was comprehensively investigated in vitro. Then, we identified that miR‐129‐5p reduced *CAMK2N1* expression, which was confirmed by dual luciferase reporter assay. Finally, we proved that miR‐129‐5p promotes docetaxel‐resistance while co‐expression of *CAMK2N1* significantly rescued this phenotype. We thus provided evidence that *CAMK2N1* was the target of miR‐129‐5p, which has potential to become a promising therapeutic target for the treatment of DR PCa.

The overexpression of *CAMK2N1* had been reduced in the progression of medullary thyroid cancer,^16^ colon cancers^17^ and PCa.^15^, ^18^ In the current report, we found the low expression level of *CAMK2N1* in both DR PCa patient tissues and PC‐3‐DR cells, which was consistent with previous studies.^15^, ^18^ Moreover, our finding indicated that in DR PCa cells, ectopic expression of *CAMK2N1* largely reduced its proliferation, survival and growth while remarkably enhanced its docetaxel‐induced apoptosis. Our data were compatible with previous researches which revealed *CAMK2N1* has a suppressive role in CRPC.^15^, ^18^ These results substantiated that *CAMK2N1* played an important role in regulating tumour growth and also mediating various drug‐resistance in PCa.

In previous studies, researchers found miR‐129 down‐regulated in gastric cancer, colorectal cancer, gastric cancer, and liver cancer.^19^, ^20^, ^21^, ^22^, ^23^ Whereas, previous study proved that high expression level of miR‐129‐5p led to development of oesophageal cancer.^10^ In addition, Xiao et al illustrated that norcantharidin increased Beclin‐1 by regulating miR‐129‐5p, which in turn trigger autophagic cell death in PCa cells.^24^ Li et al showed that lowly expressed miR‐129‐5p inhibits growth and induces apoptosis in laryngeal carcinoma by targeting adenomatous polyposis coli.^25^ Here, we verified that miR‐129‐5p acted on *CAMK2N1* to promote proliferation, migration and invasion while demoted apoptosis, resulting in the enhanced survival rate in PC‐3‐DR cells during docetaxel treatment. This result is consistent with the work presented by Zhang et al revealed that miR‐129 overexpression enhanced MDA‐MB‐231 and MCF‐7 cell resistance to docetaxel.^12^ In contrast, some publications reported the inhibitory effect of miR‐129 on tumour growth. For instance, Karaayvaz et al found miR‐129 promoted Fluoropyrimidine‐based chemotherapy in colorectal cancer treatment.^20^ The discrepancy of the conflicted role of miR‐129 in cancer during chemotherapy could contribute to the differences of drugs as the cytotoxic activity of docetaxel is exerted by improving microtubule assembly and preventing microtube disassembly while fluorouracil acts as thymidylate synthase inhibitor blocking synthesis of the pyrimidine thymidine which is required for DNA replication.^26^, ^27^ Therefore, the promoting function of miR‐129 in chemoresistance could be highly drug‐specific, which needs further studies.

MiR‐129‐5p is a miRNA that has been rarely studied, particularly in PCa. In the current study, we discovered that *CAMK2N1* as a target of miR‐129‐5p and uncovered a novel function of miR‐129‐5p in promoting proliferation and metastasis of PC‐3‐DR cells. Nevertheless, some limitations also existed in this report which should be taken into consideration. For instance, though, we identified miR‐129‐5p as a direct regulator of *CAMK2N1* protein translation in vitro, in vivo assays are still needed to further confirm the biological function of miR‐129‐5p/*CAMK2N1* axis. Besides, we found that miR‐129‐3p also had a targeting relationship with *CAMK2N1* and that their targeting regions were conserved too. So, miR‐129‐3p was also a factor with significant research value, and we would further study its effects on PCa. Nevertheless, conclusion can be drawn from our studies that the miR‐129‐5p/CAMK2N1 axis has crucial molecular and cellular functions in DR PCa cells, which provide a new therapeutic strategy for future researches.

Furthermore, CAMK2N1 caused down‐regulation of MEK/ERK activity and up‐regulation of p27 protein, which regulates the cell cycle progression of colon cancer cells,^13^, ^14^ and induced apoptosis regulatory kinases including Bax/Bcl2, caspase4, caspase7.^15^ These studies suggested how CAMK2N1 inhibited tumourigenesis via regulating cell cycle and improving cell apoptosis.

In conclusion, *CAMK2N1* was down‐regulated in PC‐3‐DR cells, as a modulator for docetaxel sensitivity. MiR‐129‐5p stimulated proliferation and progression of PC‐3‐DR cells during docetaxel treatment through targeting *CAMK2N1*. Our finding suggested miR‐129‐5p might provide a potential therapy target to CRPC in the future.

## CONFLICT OF INTEREST

The authors declare that they have no competing interests.

## AUTHOR CONTRIBUTION

Contributing to the conception and design: Cheng Wu, Chunqing Miao, Ping'an Chang, Qingsheng Tang; Analyzing and interpreting data: Chunqing Miao, Xunrong Zhou, Pengshan Xi; Drafting the article: Cheng Wu, Chunqing Miao; Revising it critically for important intellectual content: Haodong Ni, Lixin Hua; Approving the final version to be published: all authors.

## ETHICS APPROVAL

The study was approved by the Ethics Boards of the First Affiliated Hospital of Nanjing Medical University.
