# Supplementary material for: MiR‐129‐5p promotes docetaxel resistance in prostate cancer by down‐regulating CAMK2N1 expression
Source: J Cell Mol Med. 2019 Dec 26;24(3):2098–108. doi: 10.1111/jcmm.14050 (PMC7011149; doi:10.1111/jcmm.14050)
Supplement: Supplementary file 1 [file JCMM-24-2098-s001.docx]

**Table S1** Potential miRNAs of CAMK2N1 with databases of microRNA, miRDB, miRBase, RNA22v2.0 and TargetScan Human 7.1

| **microRNA** | **PicTar** | **miRDB** | **RNA22v2.0** | **TargetScanHuman 7.1** |
| --- | --- | --- | --- | --- |
| hsa-miR-129-5p | has-miR-372 | hsa-miR-6867-5p | hsa-let-7a-2-3p | hsa-miR-96-5p |
| hsa-miR-590-3p | has-miR-20 | hsa-miR-6719-3p | hsa-let-7b-5p | hsa-miR-183-5p.2 |
| hsa-miR-96 | has-miR-93 | hsa-miR-3123 | hsa-let-7c-5p | hsa-miR-17-5p |
| hsa-miR-30a | has-miR-302d | hsa-miR-493-5p | hsa-let-7d-5p | hsa-miR-302-3p |
| hsa-miR-30d | has-miR-302b | hsa-miR-183-3p | hsa-let-7e-5p | hsa-miR-182-5p |
| hsa-miR-30e | has-miR-129 | hsa-miR-6873-5p | hsa-let-7f-5p | hsa-miR-187-3p |
| hsa-miR-302e | | hsa-miR-3651 | hsa-let-7g-3p | hsa-miR-129-3p |
| hsa-miR-106b | | hsa-miR-382-3p | hsa-let-7i-3p | hsa-miR-150-5p |
| hsa-miR-20a |  | hsa-miR-4684-3p | hsa-let-7i-5p | hsa-miR-140-5p |
| hsa-miR-93 |  | hsa-miR-4311 | hsa-miR-103a-3p | hsa-miR-22-3p |
| hsa-miR-519d | | hsa-miR-513c-3p | hsa-miR-103b | hsa-miR-139-5p |
| hsa-miR-520c-3p | | hsa-miR-96-5p | hsa-miR-105-3p | hsa-miR-365-3p |
| hsa-miR-520a-3p | | hsa-miR-3160-5p | hsa-miR-105-5p | hsa-miR-7-5p |
| hsa-miR-520d-3p | | hsa-miR-1271-5p | hsa-miR-106a-3p | hsa-miR-199-5p |
| hsa-miR-302a | | hsa-miR-522-3p | hsa-miR-106b-3p | hsa-miR-153-3p |
| hsa-miR-302c | | hsa-miR-526b-3p | hsa-miR-107 | hsa-miR-24-3p |
| hsa-miR-302d | | hsa-miR-6768-5p | hsa-miR-1179 | hsa-miR-183-5p.1 |
| hsa-miR-372 |  | hsa-miR-627-3p | hsa-miR-1180-3p | hsa-miR-205-5p |
| hsa-miR-373 |  |  | hsa-miR-1182 | hsa-miR-33-5p |
| hsa-miR-182 |  |  | hsa-miR-1183 | hsa-miR-1-3p |
| hsa-miR-342-3p | |  | hsa-miR-1184 | hsa-miR-192-5p |
| hsa-miR-377 |  |  | hsa-miR-1185-5p | hsa-miR-375 |
| hsa-miR-22 |  |  | hsa-miR-1193 | hsa-miR-202-5p |
| hsa-miR-23b |  |  | hsa-miR-1199-3p | hsa-miR-520-3p |
| hsa-miR-422a | |  | hsa-miR-1199-5p | hsa-miR-211-5p |
| hsa-miR-320c | |  | hsa-miR-1202 | hsa-miR-506-3p |
|  | |  | … |  |

RNA22v2.0 predicted result had 1304 targets of CAMK2N1 and this table just presented the first 26 targets; “…” means the last all targets of CAMK2N1 with RNA22v2.0.
